# Different Timing Features in Brain Processing of Core and Moral Disgust Pictures: An Event-Related Potentials Study

**DOI:** 10.1371/journal.pone.0128531

**Published:** 2015-05-26

**Authors:** Xiangyi Zhang, Qi Guo, Youxue Zhang, Liandi Lou, Daoqun Ding

**Affiliations:** 1 Key Laboratory for Cognition and Human Behavior of Hunan Province, Department of Psychology, Hunan Normal University, Changsha, China; 2 School of Psychology, Liaoning Normal University, Dalian, China; 3 School of Life Science and Technology, University of Electronic Science and Technology of China, Chengdu, China; 4 School of Psychology and Cognitive Science, East China Normal University, Shanghai, China; Southwest University, CHINA

## Abstract

Disgust, an emotion motivating withdrawal from offensive stimuli, protects us from the risk of biological pathogens and sociomoral violations. Homogeneity of its two types, namely, core and moral disgust has been under intensive debate. To examine the dynamic relationship between them, we recorded event-related potentials (ERPs) for core disgust, moral disgust and neutral pictures while participants performed a modified oddball task. ERP analysis revealed that N1 and P2 amplitudes were largest for the core disgust pictures, indicating automatic processing of the core disgust-evoking pictures. N2 amplitudes were higher for pictures evoking moral disgust relative to core disgust and neutral pictures, reflecting a violation of social norms. The core disgust pictures elicited larger P3 and late positive potential (LPP) amplitudes in comparison with the moral disgust pictures which, in turn, elicited larger P3 and LPP amplitudes when compared to the neutral pictures. Taken together, these findings indicated that core and moral disgust pictures elicited different neural activities at various stages of information processing, which provided supporting evidence for the heterogeneity of disgust.

## Introduction

Disgust is a basic human emotion that functions to protect us from disease [1]. It is thought to have originated in oral rejection to toxic or unpleasant-tasting substances [2]. With the evolution of society, the core rejection impulse of disgust has expanded into the social-moral domains [3]. The disgust reaction triggered by contaminated food, body products (e.g., feces, vomit), specific animals (e.g., maggots, cockroaches), and other physical materials is referred to as core or physical disgust [4]. On the other hand, the disgust reaction elicited by socio-moral transgressions is referred to as moral disgust, which is crucial for maintenance of social norms [4–6]. More specifically, there are two types of moral disgust: sexual immoral behaviors (e.g., incest, bestiality, pedophilia, masturbation, prostitution), and non-sexual immoral behaviors (e.g., cheat, steal, and murder) (for a review, see [5,7,8]).

Recent studies have investigated the relationship between core disgust and moral disgust. Some researchers have suggested that core and moral disgust share a common evolutionary origin, which is referred to as an oral rejection impulse triggered by poisonous or unpleasant-tasting substances. Thus, they considered the two subtypes of disgust as fundamentally the same construct [2,9,10]. For example, Chapman and colleagues found that gustatory distaste, core disgust, and moral disgust activated similar facial muscle activities. As a result, they hypothesized that moral transgressions elicited the same response as core disgust evocators and unpleasant tastes [10]. Moreover, some studies also have revealed that immoral behavior can reduce our appetite, and even elicit nausea [11,12]. To summarize, these findings seem to indicate that core and moral disgust are homogeneous.

Other researchers, however, have believed that disgust is a heterogeneous emotion consisting of multiple subtypes with distinct characteristics [7,8,13,14]. For example, Simpson and colleagues found that the self-reported emotional response to core disgust elicitors decreased, whereas the response to moral disgust elicitors was intensified, over time. Furthermore, females and males showed similar disgust response to moral disgust elicitors, but the levels of disgust response to core elicitors were higher in females than in males [13]. Moreover, a recent study [14] showed that both physical and moral elicitors could induce disgust. However, physical elicitors mainly evoked a subjective feeling of dirtiness and the moral elicitors induced more feelings of indignation and contempt. Physical disgust intensified the activity of the parasympathetic nervous system, as assessed by electrocardiogram, and there was no concurrency for heart rate (HR), whereas moral disgust evocators intensified autonomic imbalance and heart rate but diminished vagal tone [14]. In sum, these studies have revealed that core (or physical) and moral disgust represent different emotional constructs.

Some neuroimaging studies have shown that core and moral disgust elicitors activated partially overlapping but distinct neural substrates [7,15,16]. For example, Borg and colleagues found that neural regions, including the basal ganglia, amygdala, thalamus, parahippocampal gyrus, and dorsal anterior cingulate, could be activated by both core and moral disgust. Specifically, moral disgust was associated with greater activation in the precuneus, bilateral temporo-parietal junction, temporal poles and medial prefrontal cortex, whereas core disgust was more strongly associated with the left fusiform gyrus, left amygdala, precuneus/superior parietal lobule, and bilateral lingual gyrus, with the addition of the frontal lobes [7].

As described above, there has been a debate regarding the relationship between core and moral disgust. Similarities as well as differences between the two types of disgust have been found using various measures such as subjective self-report [13], facial EMG [10], electrocardiogram [14], and fMRI measures [7,15]. However, there were limited researches investigating the dynamic relationship between core and moral disgust. More recently, Luo and colleagues [17] investigated the temporal features of processing core and moral disgust words during a lexical decision task. Although the N320 and N400 were modulated by the processing of both core and moral disgust words, the early posterior negative (EPN) was only sensitive to the processing of core disgust words, which reflected that the core disgust words could be classified and attended to at the early lexical access stage as compared to the moral disgust words [17]. However, Yang and colleagues [18,19] employed the abstract statements to describe behaviors related to physical disgust and moral violation, and found that moral disgust stimuli were processed more rapidly than physical disgust stimuli [18,19]. They argued that the inconsistent findings involving the early time differences in neural activity might be due to the different stimuli employed in these studies. Specifically, the moral disgust words used in Luo and colleagues’ study might be easily understood as verbs, whereas the core disgust words were nouns [19]. Moreover, Marzillier and Davey [20] suggested that there was a limitation to induce disgust by linguistic materials, whose presentation required subjects to imagine relevant visual scenarios [20]. Additionally, relative to emotional linguistic stimuli, the affective picture stimuli were more arousing, and would induce more genuine emotional reactions [20–24]. Considering that the spatiotemporal features of processing core and moral disgust stimuli need further clarification by other types of experimental stimuli, the present study aimed to investigate the temporal dynamics of processing pictorial stimuli evoking core and moral disgust. In order to avoid the contamination of the relevance-for-task effect reported by previous studies [25,26], a modified oddball task was used in the current study. Subjects were informed to differentiate the standard and deviant stimuli by pressing corresponding keys, ignoring the emotional valence of the deviants [27,28].

ERP measure has been frequently used to investigate the time course of emotional information processing [29–37]. For example, negative stimuli elicited an enhanced N1 amplitude relative to neutral stimuli [38,39], which suggests that negative stimuli attract attention in the early sensory processing stage [31,34,38–41]. The P2 is larger for negative pictures as compared to both the positive and neutral pictures and has been interpreted as reflecting attention is automatically oriented to negative information [27,30,33]. Moreover, Feng and colleagues [42] showed that erotic pictures also elicited larger P2 amplitudes than non-erotic negative, positive, and neutral pictures [42]. Previous studies have revealed that P3 (or LPP) is sensitive to the response of decisional processes, the cognitive evaluation of stimulus meanings, and the allocation of attentional resources [27,43,44]. Specifically, the emotional stimuli usually elicited more pronounced P3 (or LPP) amplitude when compared to neutral stimuli [38, 45–47].

Previous studies showed that core disgust stimuli, due to its salience, could be more quickly classified and attended to as compared to moral disgust and neutral stimuli at the early processing stage [17]. Core disgust was partly associated with the perceptual experience, and did not involve higher order cognitive processes [17]. Consequently, we hypothesized that core disgust pictures would elicit enhanced N1 and P2 amplitudes than moral disgust and neutral pictures in early processing stages. In contrast, the activation of moral disgust depended on complex social appraisal and judgment [48,49]. The neuroimaging studies also showed that moral disgust could activate brain regions related to social evaluation [7,15]. More recently, some studies suggest that frontal N2 is sensitive to moral violation [50,51]. Thus, we expected that the processing bias for the moral disgust pictures would occur in the N2 component, and later higher-order cognitive evaluation stages such as the P3 or LPP components. Specifically, we hypothesized that moral disgust pictures would elicit larger N2, P3 or LPP amplitudes than neutral pictures. Moreover, sustained processing of the core disgust pictures, due to their emotional saliency, would also contribute to larger P3 or LPP amplitudes for core disgust than neutral pictures.

## Materials and Methods

### Participants

Eighteen healthy undergraduate students (nine men and nine women; age M ±SD = 20.86 ±1.76; age range of 18–24) were recruited in this study. All participants were right-handed, and had normal or corrected-to-normal visual acuity. The participants did not have any physical or mental illness. This experiment was approved by the review board for human subjects of Hunan Normal University in China, and the written informed consent was obtained from all participants prior to the experiment. Participants were paid ¥50.

### Stimuli

The materials consisted of 30 deviant stimuli pictures (10 core disgust, 10 moral disgust, 10 neutral) and one standard picture (a chair). These pictures were taken from the International Affective Picture System (IAPS; [52]), Chinese Affective Picture System (CAPS; [53]) and the internet. Firstly, 616 disgusting and neutral pictures were collected based on the definition of disgust [4,54]. Then, a preliminary screening was conducted to make sure the selected pictorial stimuli had no word information. Secondly, a more rigorous screening was conducted and 180 typical pictures (60 for each kind of deviant pictures) were chosen as the candidate experimental materials for further assessment. Thirdly, Photoshop image software was used to ensure all pictures were 16 × 14 cm in size and 100 pixels per inch in resolution.

Subsequently, some representative pictures were used as examples to demonstrate how the assessment was conducted. Participants were asked to practice evaluating, for familiarization with the stimulus evaluation procedure. After participants confirmed that they were able to effectively distinguish different kinds of disgusting pictures, a formal assessment was performed for the 180 pictures. Fifty-eight participants (28 male and 30 female; age range = 17–26 years; age M ± SD = 19.81 ± 2.42), who did not participate in the ERP study, were asked to assess emotional classifications (disgust, happiness, anger, fear, sadness, surprise, and neutral). They were also asked to rate the emotional intensity (from “not at all” to “extremely”), arousal (from “very calm” to “very exciting”), as well as valence (from “very unpleasant” to “very pleasant”) of the pictures on a 9-point Likert-type scale.

Finally, 10 core disgust pictures (such as feces, vomit), 10 moral disgust pictures (such as pickpocket, abuse children), and 10 neutral pictures (such as basin, clock) were chosen based on the following criteria [17]: (1) For a picture considered to be disgusting, it must be placed into the “disgust” category by more than 95% of the participants, and had a valence rating lower than 4 and an arousal rating higher than 6. Because sexual-related pictures would activate additional brain areas [7], these pictures were excluded in the present study; (2) disgust pictures related to contaminated food, bodily secretions (vomit, feces), and body parts were classified as core disgust pictures, and those involving socio-moral violations were classified as moral disgust pictures [4,7,13,54]; (3) the emotional intensity rating of disgust pictures was higher than 7; (4) those pictures with valence ratings between 4 and 5 were categorized as neutral pictures.

Core and moral disgust pictures were selected in order to ensure that there were no significant differences in emotional intensity, *F*(1,18) = 2.47, *p* = 0.13 (M ± SD, core: 7.60 ± 0.20, moral: 7.44 ± 0.24), arousal, *F*(1,18) = 2.85, *p* = 0.11 (M ± SD, core: 7.47 ± 0.22, moral: 7.32 ± 0.17), or valence, *F*(1,18) = 2.33, *p* = 0.14 (core: 2.82 ± 0.20, moral: 2.98 ± 0.26). However, core disgust and neutral pictures differed significantly in arousal, *F*(1,18) = 2899.24, *p* < 0.001 (core: 7.47 ± 0.22, neutral: 3.25 ± 0.12), and valence, *F*(1,18) = 520.36, *p* < 0.001 (core: 2.82 ± 0.20, neutral: 4.54 ± 0.12) ratings. Moreover, moral disgust and neutral pictures also differed significantly in arousal, *F*(1,18) = 3730.33, *p* < 0.001 (moral: 7.32 ± 0.17, neutral: 3.25 ± 0.12), and valence, *F*(1,18) = 297.31, *p* < 0.001 (moral: 2.98 ± 0.26, neutral: 4.54 ± 0.12) ratings.

### Procedure

A modified oddball task was adopted in this study (for a review, see [55, 56]). It consisted of four blocks, and within each block, there were 100 trials consisting of 70 standard and 30 deviant stimuli trials. A chair picture served as the standard stimulus and 30 images (10 core disgust, 10 moral disgust and 10 neutral images) as the deviant stimuli. The stimuli were presented in a random order in each block. After each testing block, the participants were allowed to take a rest for two minutes.

**Fig 1:**
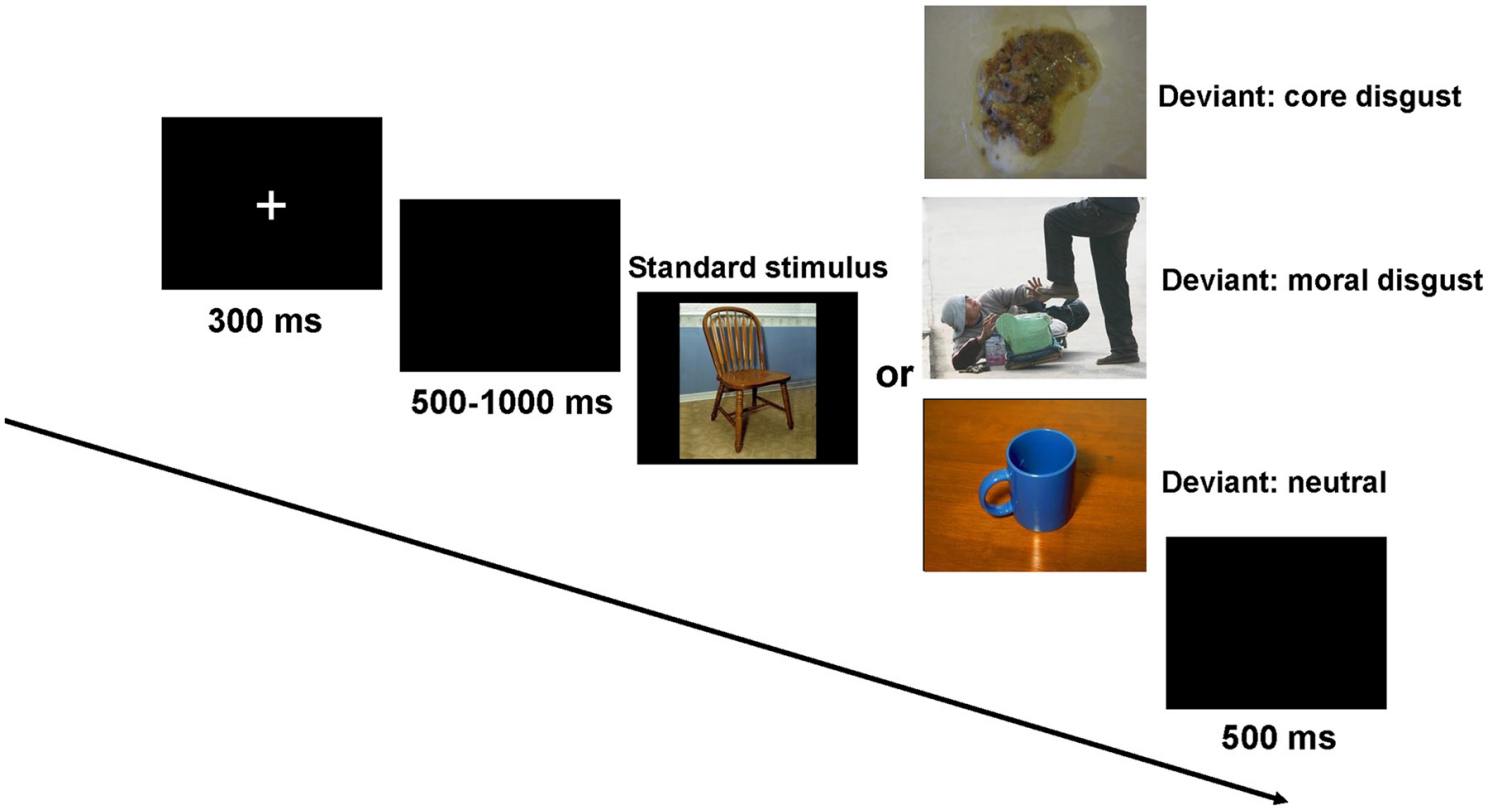
The schematic illustration of the experimental procedure. Core disgust: vomit. Moral disgust: a bad antisocial action, a person stamping on a disabled beggar. Picture presentation was terminated by a key pressing or when 1000 ms was elapsed.

Participants were seated approximately 70 cm away from a computer screen. They were informed that the aim of this study was to investigate picture processing. Participants performed 10 practice trials to be familiar with the experimental task, and then the formal experiment and EEG recording began. Each trial was started with a small white cross for 300 ms presentation on the black computer screen. Then, a black blank screen was presented for a duration that varied randomly from 500 to 1000 ms. Subsequently, the standard or deviant stimulus was presented for 1000 ms, during which half of the participants were required to press the “F” key as accurately and quickly as possible with their left index finger when the standard stimulus was presented and to press the “J” key with their right index finger when the deviant stimulus was presented. For the other half, the response pattern was reversed. Once a response was made or the picture was presented for 1000 ms, the picture would disappear. The trial ended with a blank screen whose presentation was 500 ms (see Fig 1).

### ERP recording

Electroencephalogram (EEG) activity was continuously recorded from 64 scalp sites using Ag/AgCl electrodes according to the international 10–20 system, with an online reference to the left mastoid and off-line re-referencing to the average of the left and right mastoids. The horizontal electrooculogram (EOG) was recorded from electrodes placed at 1.5 cm lateral to the left and right external canthi, and vertical EOG was recorded from electrodes placed above and below the left eye. All electrode impedances were below 5 kΩ. The EEG and EOG were amplified using a 0.05–100 Hz band pass and the sampling rate was 500 Hz/channel. Vision Analyzer software used an automatic ocular correction procedure to eliminate EOG artifacts, and a band pass filter was used (high-pass cutoff: 0.01Hz, slope: 24 dB/oct; low-pass cutoff: 30 Hz, slope: 24 dB/oct). Trials with a mean EOG voltage exceeding ±80 μV were excluded from the average ERPs.

### Data measurements and analyses

We mainly analyzed ERPs elicited by core disgust, moral disgust, and neutral deviant stimuli. The averaged epoch for ERPs was 1200 ms, including a 200 ms pre-stimulus baseline. The peak amplitudes and latencies of N1 (70–130 ms), P2 (140–210 ms), N2 (210–290 ms), P3(350–500 ms) components and the average amplitudes of LPP (600-900ms) component were investigated in their respective time window.

According to the grand-averaged ERPs’ topographical maps and the reports from previous studies [30, 31, 39], 15 electrode sites were selected, namely, F3, F1, Fz, F2, F4, FC3, FC1, FCz, FC2, FC4, C3, C1, C_Z_, C2, and C4 for statistical analysis of the N1, P2 and N2 components. The P3 and LPP were analyzed at the following 25 electrode sites: F3, F1, Fz, F2, F4, FC3, FC1, FCz, FC2, FC4, C3, C1, C_Z_, C2, C4, CP3, CP1, CP_Z_, CP2, CP4, P3, P1, P_Z_, P2, P4.

Analysis of variance (ANOVAs) was conducted with three factors: stimulus types (three levels: core disgust, moral disgust, and neutral), laterality (five levels: left, midline-left, midline, midline-right, and right), and frontality (three levels for N1, P2 and N2 components: frontal, frontocentral and central; five levels for P3 and LPP: frontal, frontocentral, central, centroparietal, and parietal). The Greenhouse—Geisser correction was adopted when the spherical assumption was violated.

## Results

### Behavioral results

An ANOVA was conducted on the reaction times (RTs) and accuracy with stimulus type as a within-subject variable, respectively. For RT, a highly significant main effect of stimulus type was observed [*F*(2,34) = 14.53, *p* < 0.001, *η*_*p*_^*2*^ = 0.46]. Post hoc paired t tests with bonferroni-holm correction revealed that RT for core disgust pictures was faster than for moral disgust pictures [*t*(17) = −2.72, *p* = 0.028] and neutral pictures [*t*(17) = −5.22, *p* < 0.001] (see Table 1). Moreover, the reaction to moral disgust pictures was also faster than the neutral pictures [*t*(17) = −2.76, *p* = 0.039]. For accuracy, a highly significant main effect of stimulus type was also observed, similarly, [*F*(2,34) = 11.93, *p* < 0.001, *η*_*p*_^*2*^ = 0.41]. Further paired t tests indicated that the accuracy for core disgust pictures was higher than for moral disgust pictures [*t*(17) = 2.72, *p* = 0.03] and neutral pictures [*t*(17) = 4.90, *p* < 0.001]. Additionally, accuracy for moral disgust pictures was also higher than for the neutral pictures [*t*(17) = 2.26, *p* = 0.037].

**Table 1:** The reaction times and accuracies for three types of stimuli (*M±SD*).

	Reaction time (ms)	Accuracy
**Core disgust**	541.81±57.06	0.99±0.01
**Moral disgust**	551.37±48.73	0.97±0.04
**Neutral**	562.27±49.91	0.95±0.04

### ERP results

#### N1

**Fig 2:**
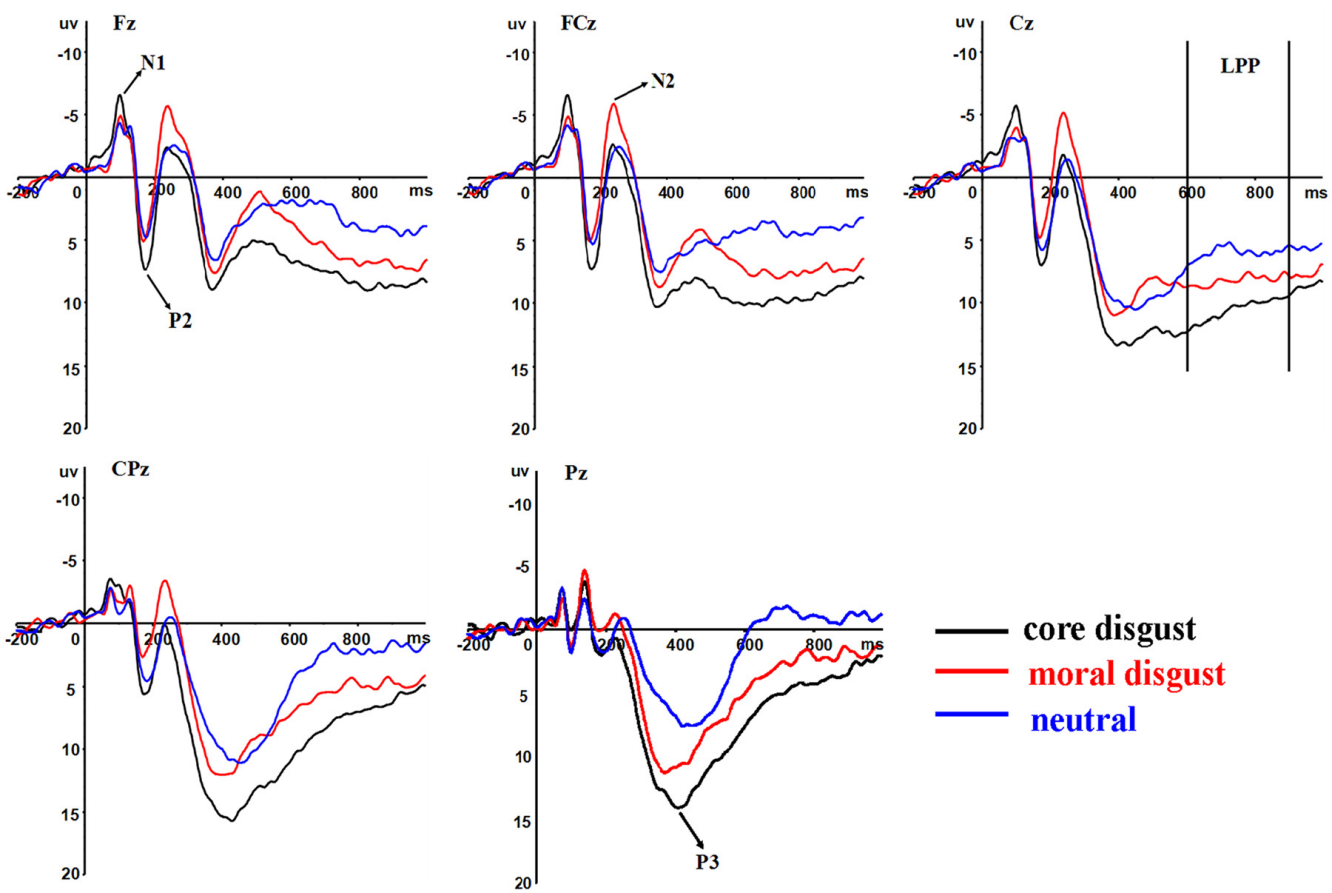
Stimulus-locked grand average ERP waveforms. Grand average ERP waveforms recorded from Fz, FCz, Cz, CPz and Pz in response to core disgust (black lines), moral disgust (red lines) and neutral (blue lines) pictures.

**Fig 3:**
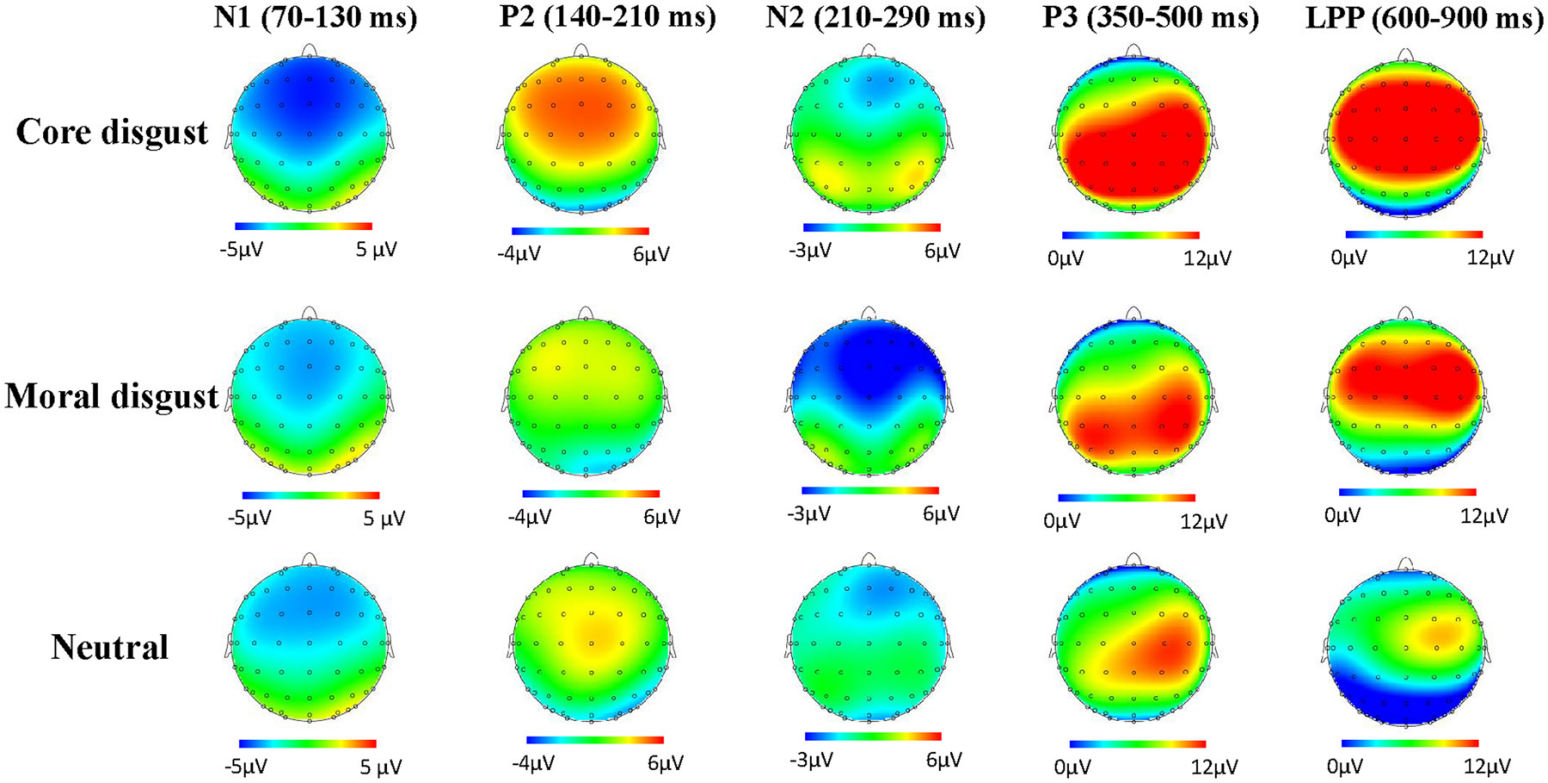
Topographical maps of voltage amplitudes of N1, P2, N2, P3 and LPP across three conditions.

The ANOVA for amplitudes of N1 revealed significant main effects of picture type [*F*(2,34) = 6.01, *p* = 0.008, *η*_*p*_^*2*^ = 0.26], frontality [*F*(2,34) = 18.19, *p* < 0.001, *η*_*p*_^*2*^ = 0.52] and laterality [*F*(4,68) = 6.58, *p* = 0.002; *η*_*p*_^*2*^ = 0.28]. There were no other main effects and no significant interaction effects for this component (*p*_*s*_ > 0.1) (see Table 2). Post hoc paired t tests with bonferroni-holm correction (the same afterwards) revealed that core disgust pictures (-7.21 μV) elicited larger N1 amplitudes than moral disgust pictures [-5.81 μV, *t*(17) = -2.82, *p* = 0.024] and neutral pictures [-5.71 μV, *t*(17) = -3.72, *p* = 0.006] and no difference was observed between moral disgust and neutral pictures [*t*(17) = -0.2, *p* = 0.84] (Fig 2). Moreover, the frontal (-6.73μV) and frontal-central (-6.47 μV) regions showed larger N1 amplitudes in comparison with central region (-5.53 μV, *p*_*s*_ < 0.001). The midline region (-6.8 μV) exhibited larger N1 amplitudes than the left (-6μV) and right (-5.76 μV) lateralized regions *(p*_*s*_ < 0.05) (Fig 3). Additionally, no significant effects were observed for N1 latencies (*p*_*s*_ > 0.1).

**Table 2:** Results of ANOVA for the amplitudes of N1, P2, N2, P3 and LPP components.

	picture type		frontality		laterality		picture type* frontality		picture type* laterality		picture type* laterality* frontality	
	*F*	*P*	*F*	*P*	*F*	*P*	*F*	*P*	*F*	*P*	*F*	*P*
**N1**	6.01	0.008	18.19	0.0001	6.58	0.002	1.06	0.37	1.49	0.22	0.56	0.71
**P2**	7.12	0.003	1.4	0.26	2.47	0.11	1.67	0.19	2.03	0.12	0.99	0.39
**N2**	5.9	0.009	2.96	0.093	5.75	0.006	2.15	0.12	1.56	0.2	1.03	0.4
**P3**	9.29	0.001	5.18	0.028	8.55	0.001	7.83	0.0001	1.98	0.11	0.92	0.46
**LPP**	31.7	0.0001	20.65	0.0001	2.29	0.11	0.89	0.47	3.37	0.027	1.52	0.19

#### P2

The ANOVA for amplitudes of P2 demonstrated that the main effect of picture type were significant [*F*(2,34) = 7.12, *p* = 0.003, *η*_*p*_^*2*^ = 0.3] (see Table 2). Post hoc paired t tests showed that core disgust pictures (8.2 μV) elicited larger P2 amplitudes than moral disgust [6.15 μV, *t*(17) = 2.85, *p* = 0.022] and neutral pictures [5.78 μV, *t*(17) = 3.82, *p* = 0.003]. No difference reached significance for P2 amplitudes between moral disgust and neutral picture conditions [*t*(17) = 0.52, *ns*] (Fig 2). No other effects approached significance on P2 amplitudes (*p*_*s*_ > 0.1). In addition, the main effect of picture type for P2 latencies was significant [*F*(2,34) = 6.02, *p* = 0.007, *η*_*p*_^*2*^ = 0.26]. The latencies for moral disgust pictures were shorter than for neutral pictures (*p* = 0.01), but no significant difference between core and moral disgust pictures (*p* > 0.05).

#### N2

The ANOVA for the N2 amplitudes demonstrated significant main effects of picture type [*F*(2,34) = 5.9, *p* = 0.009, *η*_*p*_^*2*^ = 0.26] and laterality [*F*(4,68) = 5.75, *p* = 0.006, *η*_*p*_^*2*^ = 0.25] (see Table 2 and Fig 2). Post hoc paired t tests revealed that moral disgust pictures (-5.49 μV) elicited enhanced N2 amplitudes than core disgust [-2.53 μV, *t*(17) = -3.09, *p* = 0.021] and neutral pictures [-3.54 μV, *t*(17) = -2.04, *p* = 0.058, uncorrected], and no difference was observed between core disgust and neutral pictures [*t*(17) = 1.49, *p* = 0.16]. The midline region (-4.8 μV) displayed more pronounced N2 amplitudes as compared to the left (-3.03 μV) and right (-3.62 μV) lateralized regions *(p*_*s*_ < 0.05). In addition, no significant effects were observed for N2 latencies (*p*_*s*_ > 0.1).

#### P3

The ANOVA for P3 amplitudes demonstrated significant main effects of picture type [*F*(2,34) = 9.29, *p* = 0.001, *η*_*p*_^*2*^ = 0.35], frontality [*F*(4, 68) = 5.18, *p* = 0.028, *η*_*p*_^*2*^ = 0.23] and laterality [*F*(4,68) = 8.55, *p* = 0.001, *η*_*p*_^*2*^ = 0.34], besides, a significant interaction between frontality and picture type [*F*(8,136) = 7.83, *p* < 0.001, *η*_*p*_^*2*^ = 0.32] (see Table 2 and Fig 2). P3 amplitudes were largest at centraparietal site (14.88 μV, *p* = 0.004) and smallest at frontal site (9.93μV). The scalp midline (12.68 μV, *p* = 0.028) and right lateralized (13.61 μV, *p* = 0.004) regions displayed larger P3 amplitudes as compared to the left lateralized (11.52 μV) regions (Fig 3). Breaking down the frontality by picture type interaction showed that core disgust pictures elicited larger P3 amplitudes than moral disgust pictures (*p*<0.001) which, in turn, elicited enhanced P3 amplitudes compared to neutral pictures (*p*<0.001) only at central-parietal [*F*(2,34) = 14.88, *p* < 0.001, *η*_*p*_^*2*^ = 0.47] and parietal sites [*F*(2,34) = 24.19, *p* < 0.001, *η*_*p*_^*2*^ = 0.59], but no such effect was observed at frontal, frontal-central and central sites. In addition, no significant effects were observed for P3 latencies (*p*_*s*_ > 0.1).

#### Late Positive Potentials (LPP)

In the 600–900 ms, the ANOVA for LPP amplitudes demonstrated significant main effects of picture type [*F*(2,34) = 31.7, *p* < 0.001, *η*_*p*_^*2*^ = 0.65] and frontality [*F*(4,68) = 20.65, *p* < 0.001, *η*_*p*_^*2*^ = 0.55] (Fig 2). Post hoc paired t tests showed that the amplitudes were larger at central (7.78 μV, *p* < 0.001) and frontal-central (7.61 μV, *p* < 0.001) sites than at parietal (1.96 μV) sites (Fig 3). Most importantly, core disgust pictures [7.96 μV, *t*(17) = 2.96, *p* = 0.018] elicited larger positive deflections than moral disgust pictures (6.21μV) which, in turn, elicited enhanced amplitudes when compared to neutral pictures [2.85 μV, *t*(17) = 5.55, *p* < 0.001]. Moreover, the laterality by picture type interaction was significant [*F*(8,136) = 3.37, *p* = 0.027, *η*_*p*_^*2*^ = 0.17]. The simple effect analysis showed that the core and moral disgust conditions elicited larger LPP amplitudes than the neutral condition at all regions (*ps* < 0.001). However, the core disgust condition elicited larger LPP amplitudes than the moral disgust condition only at midline sites [*t*(17) = 3.62, *p* = 0.006].

#### The timing of emotion effects for core and moral disgust pictures

In order to strengthen our conclusion that the emotion effect for the core disgust (N1 and P2) occurred earlier than that for the moral disgust (N2), a timing analysis was conducted. First, the amplitude data were collapsed across the 15 frontal, frontal-central and central sites for the N1, P2 and N2 components. Then, an ANOVA was performed with timing (3 levels: N1, P2 and N2) and stimulus type (3 levels: core disgust, moral disgust and neutral stimuli) as factors [57]. The results demonstrated a significant interaction effect between timing and stimulus type [*F*(4, 68) = 13.34, *p* < 0.0001, *η*_*p*_^*2*^ = 0.44]. Core disgust pictures elicited a significant emotion effect in N1 [*t*(17) = -3.72, *p* = 0.006] and P2 [*t*(17) = 3.82, *p* = 0.003] stages; while the emotion effect for moral disgust pictures approached significance only in the N2 stage [*t*(17) = -2.04, *p* = 0.058]. Thus, the timing analysis strengthened our conclusion of faster, more automatic emotion effect for core disgust relative to moral disgust.

## Discussion

Using ERP measures, the current study aimed to investigate the time courses of information processing associated with disgust pictures. The behavioral results demonstrated that core disgust pictures elicited faster and more accurate responses relative to the moral disgust pictures which, in turn, induced faster and more accurate responses when compared to the neutral pictures. Meanwhile, the ERP results showed that core disgust pictures elicited enhanced N1 and P2 amplitudes than the moral disgust and neutral pictures did. Furthermore, moral disgust pictures generated enhanced N2 amplitudes than the core disgust and neutral pictures did. Most importantly, core disgust pictures evoked enhanced P3 and LPP amplitudes than did moral disgust pictures which, in turn, evoked enhanced P3 and LPP amplitudes when compared to neutral pictures. Thus, the present study suggests that the processing of the core and moral disgust pictures may be mediated by distinct neurocognitive mechanisms.

In the current study, enhanced anterior N1 amplitudes were observed for core disgust pictures compared to moral disgust and neutral pictures. It has been suggested that anterior N1 reflects the level of attention, with higher amplitudes for attended stimuli than for non-attended stimuli [58]. More specifically, some researchers found that fearful faces induced enhanced N1 amplitudes when compared to happy and neutral faces, indicating that fearful faces preferentially and automatically attracted attention in the early information processing stage. Therefore, they argued that the emotion effect on N1 evoked by fearful faces might reflect fast and automatic detection for threatening information, which is biologically significant for individuals [34,41]. Similar to fearful facial expressions, core disgust pictures also elicited enhanced N1 amplitudes when compared to moral disgust and neutral pictures. The N1 effect evoked by core disgust pictures could be due to the fact that these stimuli may be as biologically relevant as fearful stimuli [59]. This preferential processing for core disgust pictures obviously has adaptive and evolutionary advantages, and may function to motivate pathogen avoidance [5].

In addition, larger P2 amplitudes were also observed for the core disgust pictures when compared to the moral disgust and neutral pictures. The P2 component has been considered as a neural index of automatic attention processing [30,60]. Therefore, an enhanced P2 elicited by core disgust pictures may reflect that core disgust pictures capture human attention rapidly and automatically. However, the effect for the moral disgust pictures was not observed at the early automatic processing stages, such as the N1 and P2 components. It could be because the biological importance of moral disgust pictures was far less than that of core disgust pictures, and the urgency of recognizing and analyzing moral disgust pictures was reduced relative to the core disgust stimuli [1]. However, it should be noted that the perceptual properties of emotional pictures are likely to modulate the early ERP components [61]. Thus, the different neural responses to core and moral disgust pictures in the early N1 and P2 components might due to the different physical attributes of these two types of disgust pictures.

More interestingly, a larger medial frontal N2 was observed for the moral disgust pictures than for the core disgust and neutral pictures. The frontal N2 has been interpreted as reflecting cognitive control, conflict-monitoring and expectancy violation [62–65]. Moreover, previous researches have demonstrated that the medial frontal N2 is sensitive to the violation of social rules [50, 51, 66, 67]. For example, Lahat and colleagues found that moral violations elicited larger N2 amplitudes as compared to conventional violations [50]. In addition, Wu and colleagues also revealed that an enhanced frontal negativity was observed in highly unequal offers than in moderately unequal offers during an ultimatum game [67]. In our study, moral disgust pictures were related to those behaviors that violated the moral values and social rules. Generally speaking, the violation of social norms might also evoke the emotion of anger. Thus, the larger N2 amplitudes for moral disgust stimuli might be due to the enhanced attention resource devoted to anger-related information.

After the N2, larger P3 amplitudes were observed during the 350–500 ms interval for the core and moral disgust pictures relative to the neutral pictures. In addition, the core disgust pictures also elicited larger P3 amplitudes than the moral disgust pictures did. It has been suggested that the parietal P3 reflects the processes of response decision during the visual choice reaction tasks [68–70]. Thus, the larger P3 amplitudes observed for the core disgust pictures could indicate that more cognitive resources were dedicated to response decision in the core disgust condition than in other conditions. This interpretation was supported by our behavioral findings that core disgust pictures elicited faster motor responses than did moral disgust pictures which, in turn, elicited faster motor responses relative to neutral pictures.

As expected, we observed the most prominent LPP (600–900 ms) activity over central region sites in this study. The core disgust pictures evoked enhanced LPP amplitudes than did the moral disgust pictures which, in turn, elicited enhanced LPP amplitudes than the neutral pictures did. Luo [17] found that core disgust words elicited larger LPC amplitudes than the moral disgust and neutral words did, whereas no significant differences were observed between the moral disgust and the neutral words [17]. More recently, Yang and colleagues [19] used sentences to evoke the experiences of disgust and morality, and found that the morally wrong and physically disgusting condition (the WD condition) elicited larger LPC amplitudes than the morally wrong and not physically disgusting condition (the WN condition) and the morally neutral and not disgusting condition (the NN condition). However, no significant difference existed between the WN and NN conditions. Thus, in contrast to the findings shown in linguistic materials, the current study revealed a significant difference between the moral disgust and neutral pictures in the LPP component. The present findings might reflect that moral disgust evoked by pictorial materials could be encoded more deeply and elaborately than that by linguistic materials at the late processing stage indexed by LPP. The previous studies demonstrated that the LPP could be modulated by the emotional salience of stimuli and was more enhanced in processing emotional stimuli than neutral stimuli [45,71–75]. Moreover, the LPP was also sensitive to more specific picture content within the broad categories of pleasant, neutral, and unpleasant [39,76]. For example, a previous study showed that the unpleasant pictures elicited enhanced LPP amplitudes than pleasant pictures did. Moreover, within the unpleasant pictures, the LPP amplitudes evoked by core or physical disgust pictures were more enhanced than other types of disgusting and threatening pictures [39]. More recently, Luo [77] also showed that highly negative emotional pictures elicited enhanced LPP amplitudes than did moderately negative emotional pictures which, in turn, elicited enhanced LPP amplitudes than neutral pictures did [77]. The findings that core disgust pictures evoked larger LPP amplitudes than the moral disgust pictures might reflect different emotional salience in two types of disgust stimuli. Core disgust protects the body by avoiding contact with contaminating substances in a physical environment, whereas moral disgust may protect individuals’ soul by discouraging the endorsement of immoral actions and maintaining social order in the social environment [4,18]. However, core disgust is more directly related to basic biological imperatives and is more fundamental for an individual's survival and reproduction as compared to moral disgust [4,54], which could contribute to larger LPP amplitudes for the core disgust pictures relative to the moral disgust pictures.

Additionally, it is worth noting that each experimental picture in the present study was repeated for four times. One may argue that these repetitions could lead to emotional habituation that obscures the interpretation of the differences between core and moral disgust conditions. However, previous studies revealed that negative stimuli could be resistant to habituation [78,79]. For example, Carretié and colleagues [78] found that the N1 amplitudes elicited by positive and neutral pictures were significantly decreased in final trials than in initial trials, whereas the N1 amplitudes for negative pictures did not show this effect. They further suggested that the different habituation effects between negative and positive stimuli might be attributed to the valence rather than the arousal of these stimuli [78]. A recent study explored the influence of music-induced mood on neural responses to emotional images. The findings revealed that the negative music priming enhanced rather than diminished the emotional arousal to negative images [80]. Thus, the core and moral disgust stimuli, both of which are salient in negative valence, are most likely resistant to habituation. In this regard, the ERP differences across moral and core disgust conditions were most likely a result of their different adaptive values, rather than emotional habituation.

Taken together, the present study showed that the processing of core and moral disgust pictures were mediated by different neurocognitive mechanisms. Core disgust pictures could be attended to and encoded more rapidly and automatically, as evidenced by enhanced N1 and P2 amplitudes. Moral disgust pictures elicited enhanced frontal N2 amplitudes as compared to the core disgust and neutral pictures, reflecting the violation of social norms. At later controlled and elaborative processing stages, the core disgust pictures elicited larger P3 and LPP amplitudes than did the moral disgust pictures which, in turn, evoked larger P3 and LPP amplitudes than the neutral pictures did, reflecting faster motor responses and higher motivational significance for core disgust pictures. In sum, the present study advanced our understanding of the nature of disgust, and suggested that disgust is not a unified emotion. The knowledge about the different neurocognitive systems that mediate the processing of core and moral disgust stimuli, could make better understanding of human moral behavior, and might provide neurocognitive marker to diagnose patients whose symptoms involve impairment in moral domain, such as psychopath.

## Supporting Information

S1 File Raw data of the assessment for 180 pictures, behavioral results and ERP component amplitudes and latencies. (RAR)
